# Comparative Study on the Thermal Performance of Cr-Cr_x_O_y_ and YSZ-CoNiCrAlY Coatings Exposed at 900 °C

**DOI:** 10.3390/ma14206040

**Published:** 2021-10-13

**Authors:** Markus Kiryc, Norbert Kazamer, Deniz Kurumlu, Gabriela Marginean

**Affiliations:** 1 Department of Materials Science and Testing, Westphalian University of Applied Sciences Gelsenkirchen Bocholt Recklinghausen, Neidenburger Str. 43, 45897 Gelsenkirchen, Germany; markus.kiryc@w-hs.de (M.K.); deniz.kurumlu@w-hs.de (D.K.); gabriela.marginean@w-hs.de (G.M.); 2 Westphalian Energy Institute, Westphalian University of Applied Sciences Gelsenkirchen Bocholt Recklinghausen, Neidenburger Str. 43, 45897 Gelsenkirchen, Germany

**Keywords:** thermal barrier coatings, oxidation performance, microhardness Cr coatings, atmospheric plasma spraying, yttria stabilized zirconia

## Abstract

Yttria stabilized zirconia (YSZ) thermal barrier coatings (TBCs) deposited on CoNiCrAlY oxidation protective bond coats are commonly required in temperature regimes up to 1200 °C (e.g., hot gas turbine regions) due to their superior thermal behavior and mechanical properties. For temperatures up to around 900 °C, oxidation protection can be alternatively provided by metallic-ceramic Cr-Cr_x_O_y_ coatings. For the present research, Cr-Cr_x_O_y_ atmospheric plasma sprayed (APS) and YSZ-CoNiCrAlY APS-high velocity oxy-fuel TBC coatings were deposited on a NiCr20Co18Ti substrate. The samples were isothermally heat treated at 900 °C for 10 h in an environmental atmosphere and subsequently isothermally oxidized at the same temperature for 1200 h. Investigations of the physical, chemical, and mechanical properties were performed on the as-sprayed, heat-treated, and oxidized samples. The oxidation behavior, microhardness, cohesion, and adhesion of the samples were correlated with the microstructural investigations and compared to the conventional TBC system. It could be shown that heat treating decreased the Cr-Cr_x_O_y_ coatings crack susceptibility and led to the formation of a protective thermally grown Cr oxide layer. The experimental work on the YSZ-CoNiCrAlY system revealed that the phase composition of the bond coat has a direct influence on the oxidation protection of the coating system.

## 1. Introduction

Gas turbines are commonly known as stationary or flying power engines in the energy or aviation industry [1]. These energy conversion engines differ from each other in size and in type of energy use, but the functional principle remains the same for both. The components inside of a gas turbine are subjected to temperature changes during revisions or flight operations. Working temperatures, mechanical forces, pressures, and environmental conditions are factors that must be considered for the construction and use of such equipment [2]. Therefore, a safe and reliable operation with the longest possible service life remains a major technical challenge for the industry.

The greatest possible economic benefit for manufacturers and operators can only be achieved through high performance. The efficiency of a gas turbine *η*, described by the Carnot equation [3] displayed in Equation (1), strongly depends on the process-related gradient between the cold *T_c_* and hot *T_h_* temperatures of the reservoir and thus on the inlet temperature of the gas stream that flows from the combustor into the high-pressure turbine [4].

(1) $$\eta =1-\frac{T_{c}}{T_{h}}$$

As the efficiency of the machines is improved by increasing the temperatures in the combustion chamber [5] and in order to handle working parameters that can reach up to 1750 °C, the materials need to be well-adjusted [6,7].

However, an application-optimized design with only metallic materials does not meet the requirement of a long service time any longer. For this reason, thermal barrier coatings (TBC) systems for the combustion chambers and for the blade surfaces have been continuously developed since the 1970s [4]. These TBC systems limit the temperature of the base material surface despite high turbine inlet temperatures that can reach 1500 °C and to protect the material from thermally induced oxidation, molten salt, and hot gas corrosion.

Numerous research projects dealt already with the protective effect of TBC systems on blades and vanes in gas turbine regions, following the combustion chamber and exceeding 900 °C [1,5,8,9,10,11]. In TBC systems, TBCs are deposited as top coats (TC) by means of atmospheric plasma spraying (APS) or electron beam physical vapour deposition (EB-PVD) processes commonly on MCrAlY (M = Ni, Co or NiCo) bond coats (BC). In operation, TBCs are subjected to temperature fluctuations that cause mechanical stresses. Yttria stabilized zirconia (YSZ) TBCs are known for their low thermal conductivity and thermal flexibility due to their chemical composition and microstructure. Structurally seen, APS-deposited TBCs contain a relatively high porosity and number of microcracks. These are supposed to compensate the expansion and shrinkage stresses in relation to MCrAlY BCs and thus counteract their delamination.

MCrAlY BCs, usually deposited by high velocity oxy-fuel (HVOF), improve the adhesion of the TBC through a rough surface and compensates the misfit between the coefficients of thermal expansion (CTE) of the TBC and the substrate. While exposed to high temperatures, MCrAlY coatings form a thermally grown oxide layer (TGO) on their surface. This continuously growing layer strongly affects the TBC adhesion, which depends on the TGO’s phase composition. However, a stable TGO made of α-Al_2_O_3_ is mandatory to prevent further materials oxidation and thus to protect the substrate. This may, inter alia, include barrier function to oxygen ions [12,13,14]. Due to the number and the interaction of these complex diffusion-controlled phenomena, research projects still deal with the understanding of TBC systems oxidation behavior and its failure mechanisms [12,13,14].

Since TBC systems have proven to be reliable in the industry as long-term protection, they continue to be used mainly in the first gas turbine blade stages, where ambient temperatures exceed 1000 °C. The temperature decreases towards the turbine outlet at the last stages of the high-pressure turbine and especially in the low-pressure turbine down to 900 °C and below [6]. In these temperature regions, although TBCs can be used for safety reasons, MCrAlY coating monolayer are usually applied, which provide a sufficient long-term oxidation resistance.

Alternatively to MCrAlYs, Cr-based coatings are well-known for their use in temperature regions below 900 °C in order to protect engineering components against hot corrosion [15]. Some of the most important properties of Cr_2_O_3_ are that it forms an adherent, dense, and insoluble layer below 1000 °C in an oxidizing atmosphere [16,17] and that it is suitable for applications that require wear and corrosion protection [18]. Additionally, coatings containing fine Cr_2_O_3_ exhibit superior mechanical properties [19]. Metallic additives, including NiCr, help to increase ductility and corrosion resistance. SiO_2_ serves as a carrier material for submicron metal powders in the spraying process that are necessary to obtain a crack-poor, compact, and thus gas-tight coating.

Except for thin Cr diffusion layers used against hot corrosion processes, thicker Cr-based coatings deposited chemically or galvanically are usually inappropriate for meeting the requirements of a protective coating under thermal loads. Although Cr could be used up to 1000 °C, hardness, brittleness, multiple microcracks, weak adhesion to the substrate, and low CTE limit the application of Cr-based coatings for the mentioned purpose. In previous works, Verlotski [20] tried to address some of the above-mentioned issues by varying the chemical composition stoichiometry of Cr, CrNi, and SiO_2_ APS-sprayed coatings.

The aim of the present study was to investigate the thermal behavior and oxidation performance of Cr-based coatings on Ni-based substrate at temperatures up to 900 °C. The differences concerning the oxidation resistance are outlined in comparing the obtained results with the ones of the conventional MCrAlY BCs.

## 2. Materials and Experimental Procedure

### 2.1. Materials and Coatings Deposition

To produce the Cr-based (Cr-Cr_x_O_y_) coatings, Cr powder (H.C. Starck, Goslar, Germany) was milled from an initial fraction size of –45 + 15 µm to <5 µm in order to be able to uniformly melt the particles during deposition. Some Cr_2_O_3_ particles have been partly formed through the milling of Cr. To reduce the strain stresses between the chromium particles and to promote a high ductility of the coating, the Cr material was mixed with a <5 µm sized Ni80Cr20 powder (H.C. Starck, Goslar, Germany). Finally, the Cr-NiCr powder was mixed with −100 + 50 µm-sized SiO_2_ (cristobalite) particles that mitigate the microparticles agglomeration and act as carrying material. The final feedstock powder consisted of 30–50 wt-% Cr, 5–10 wt-% NiCr, and the rest as balance of SiO_2_. The Cr-Cr_x_O_y_ coating was deposited on a NiCr20Co18Ti (Nimonic 90) substrate with an Axial III plasma-spraying equipment at the partner company Thermico Engineering GmbH, Dortmund, Germany.

The state of the art reference YSZ-CoNiCrAlY TBC system consisted of a top APS-sprayed coating obtained from a −106 + 45 µm-sized 8 wt-% Y_2_O_3_-ZrO_2_ powder (Saint-Gobain, Blois, France). The CoNi32Cr21Al8Y0.5 BC was HVOF sprayed using a −50 + 20 µm fraction-sized feedstock powder (Oerlikon-Metco, Kelsterbach, Germany) on the Nimonic 90 substrate at the company GTV Verschleißschutz GmbH, Luckenbach, Germany.

### 2.2. Heat Treatment and Oxidation

The as-sprayed Cr-Cr_x_O_y_ coatings and TBC systems have been isothermally heat treated in a Nabertherm Universal N7/H Furnace, Lilienthal, Germany in a normal atmosphere at 900 °C for 10 h in order to improve properties such as tightness, adhesion, and residual stresses. For the investigation of the long-time oxidation behavior of the coatings, the samples were subsequently exposed at the same temperature for 1200 h.

### 2.3. Characterisation

The microstructural investigation of as-sprayed and isothermally oxidized samples was performed with a Philips XL30 scanning electron microscope (SEM), Eindhoven, Netherlands while the chemical composition was analyzed using a FEI EDAX detector, Tilburg, The Netherlands. The open source ImageJ software (version 1.53j, 2021, National Institute of Health, WI, USA) was used to evaluate the degree of internal oxidation of the Cr-Cr_x_O_y_ coatings by analyzing at least five areas of 23,400 µm^2^ in different positions per sample. Microhardness indentations HV 0.1 were performed with a ZwickRoell, ZHVµM, Ulm, Germany apparatus. An average hardness was calculated from five indents per analyzed area. The crack propagation and the morphological aspects generated by Vickers indentations were investigated with a Keyence VK-X260K confocal laser scanning microscope (CLSM), Osaka, Japan.

## 3. Results and Discussions

### 3.1. Structural Characterisation

**Figure 1:**
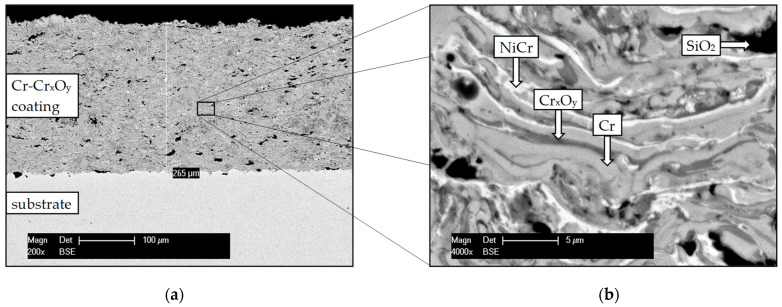
SEM/BSE cross-section micrographs of the as-sprayed Cr-Cr_x_O_y_ coating (**a**) overview, (**b**) higher magnification.

The structure and components distribution of the as-sprayed Cr-Cr_x_O_y_ coating are presented in Figure 1.

The approximately 250 µm-thick Cr-Cr_x_O_y_ coating consists of homogeneously distributed chromium (light grey), chromium oxide (dark grey), nickel chromium (bright), and silicon dioxide/cristobalite (black) phases. Cr oxides are already present in the as-sprayed coating, being formed during the fabrication of the commercially available Cr powder or even during the deposition process. Despite a reducing plasma flame during spraying, microscopical analysis shows that the formation of Cr oxide cannot be avoided during APS. This presumption is theoretically based on the Ellingham diagram, where the formation, existence, and stability of different oxides is predicted. During the atmospheric spraying process, the O_2_ partial pressure is regarded to be negligible, but sufficient to form stable Cr_2_O_3_ compounds [21]. However, a complete oxidation does not take place during spraying. The in-flight powder is covered and partially protected by the plasma during its transfer from the gun to the substrate. The formation of a purely ceramic and brittle layer of Cr oxides, with just isolated NiCr components, is therefore prevented. SiO_2_/cristobalite initially acted as a carrier powder and remained partially in a coarse granular structure trapped in the coating.

The coating has ferromagnetic properties in the as-sprayed state (as tested with a permanent magnet), assuming that metastable ferromagnetic CrO_2_ must be present in addition to the stable Cr_2_O_3_ phase (further referred as Cr_2_O_3, primary_) [22]. The CrO_2_ phase may thermodynamically originate during the spraying process by oxidation of Cr or by a more complex reduction reaction of CrO_3_ [23,24,25]. This observation will be separately investigated in the frame of further laboratory work.

The thermal performance of the Cr-Cr_x_O_y_ coatings under isothermal conditions was characterized by comparing the morphology of the as-sprayed coating with that of the heat-treated and the isothermally oxidized samples.

**Figure 2:**
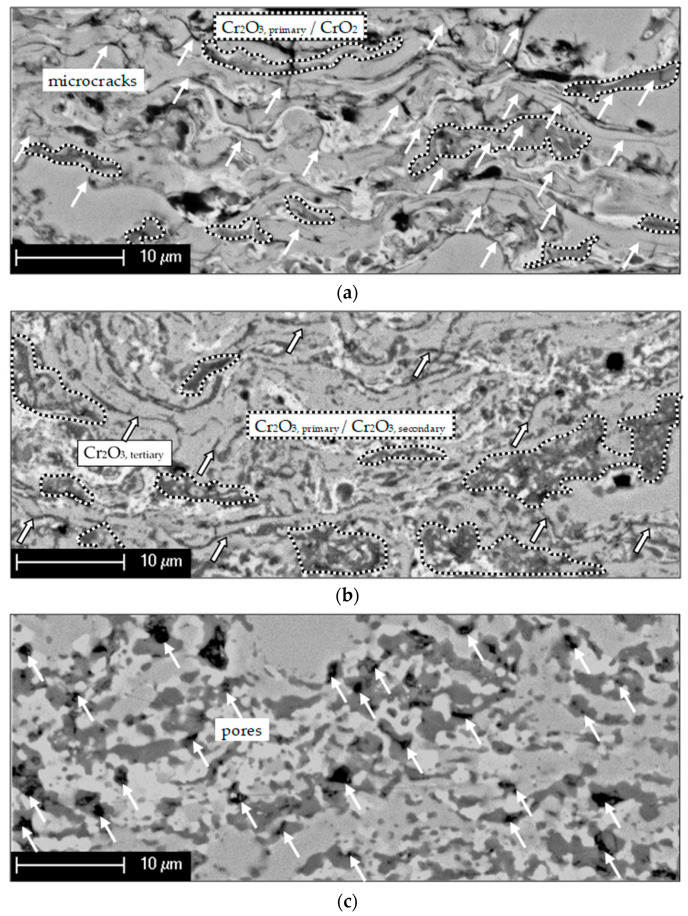
Alteration of Cr-CrxOy coating microstructures exemplarily indicated in the (**a**) as-sprayed, (**b**) heat-treated (t_900°C_ = 10 h), and (**c**) isothermally oxidized (t_900°C_ = 1200 h) states.

Figure 2 displays the morphological changes during the exposure to isothermal conditions. Several research groups reported that once the coating material is exposed to temperatures higher than 300 °C, metastable CrO_2_ phase can transform to stable Cr_2_O_3_ [23,24,25,26], here further referred to as Cr_2_O_3, secondary_.

As presented in Figure 2a, the as-sprayed coating contains numerous microcracks (indicated by white arrows), which can originate from the relaxation of shrinkage stresses of the splats during cooling. A gaseous atmosphere can penetrate directly through these microcracks or pores, and molecular oxygen reacts with the coating material due to thermally activated processes forming, especially Cr oxides. (See comparison between Figure 2a,b). The morphology of the coating, shown in Figure 2b, changed significantly during the isothermal heat treatment. The changes in the microstructure can be explained as being due to the partial oxidation of the coating material (self-healing effect, which can lead to “gas tightness”), especially in the region of the microcracks or pores forming stable Cr_2_O_3_, here referred to as Cr_2_O_3, tertiary_. A prolonged isothermal heat treatment redistributed the phases due to specific diffusion processes. As displayed in Figure 2c, such diffusion mechanisms will cause porosity inside the coating, relating to the Kirkendall effect [12], which might negatively affect the coating thermal stability.

**Figure 3:**
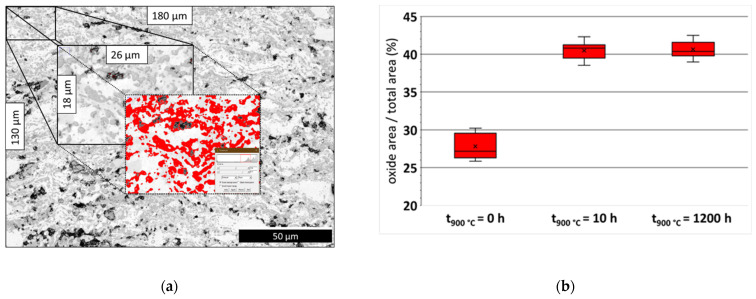
ImageJ examination of the coating in cross-section: (**a**) exemplary visualization of oxide area to total area, (**b**) statistical evaluation of results.

As observed in Figure 2c and Figure 3b, the quantity of oxide content in the coating does not change anymore, even after prolonged isothermal oxidation up to 1200 h. Differences in respect to the oxide content depending on the oxidation period can be seen in Figure 3 and are confirmed by statistically analyzing the oxide content with ImageJ-software. While the former oxide content of the as-sprayed coating has been determined to a mean value of around 27%, oxidation caused by heat treatment increased the oxide content to 40%.

**Figure 4:**
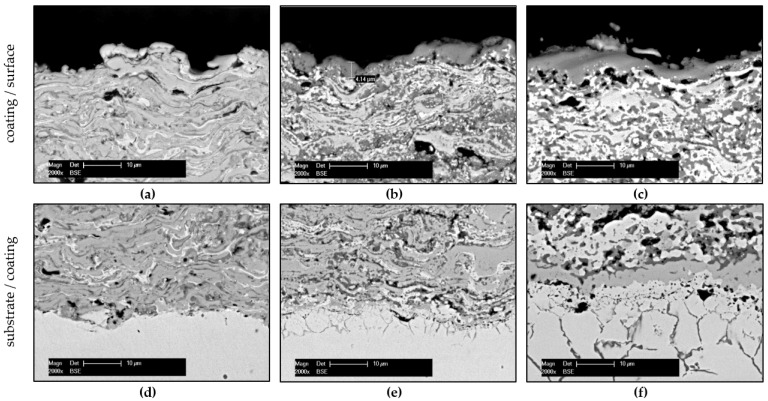
Cross-section micrographs of the Cr-Cr_x_O_y_ coating (**a**,**d**) as-sprayed, (**b**,**e**) heat-treated (t_900°C_ = 10 h), and (**c**,**f**) isothermally oxidized (t_900°C_ = 1200 h).

The structural changes at the coating surface and along the interface coating/substrate were investigated in correlation with the exposure time of the isothermal load and can be seen in Figure 4.

As shown in Figure 4b, a thermally grown Cr_2_O_3_ layer has formed on the surface of the coating after 10 h of heat treatment, contributing to the oxidation resistance over longer periods of thermal load. Since this layer exhibits a homogenous thickness distribution over the entire coating surface, further oxidation of coating material can only be caused by a kinetically delayed oxygen anion diffusion through the grown oxide layer [27]. Prolonging the oxidation led to some microstructural changes in coating morphology beneath the grown oxide layer, which exhibits similar thickness in comparison with that of the heat-treated samples, as seen in Figure 4b,c. In this case, a parabolic oxidation mechanism can be related to the outer diffusion of Cr ions, which is typically for Cr-based materials, leaving voids underneath the oxide layer [28,29]. These voids are generated due to the same Kirkendall effect mentioned before.

**Figure 5:**
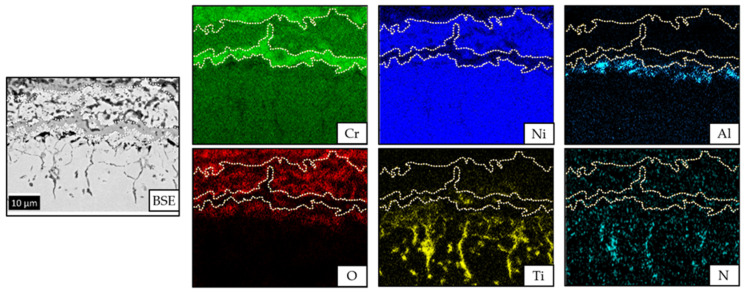
EDX-Mapping of the isothermally oxidized Cr and Ni reaction layer at the coating-substrate interface region.

**Figure 6:**
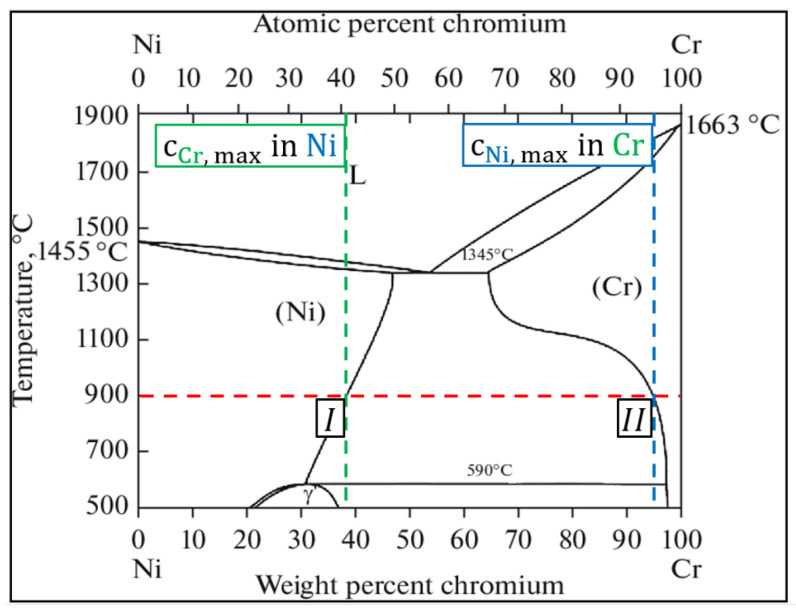
Ni-Cr alloy phase diagram, identification of the temperature-dependent solubility of elements in their solid solutions [32].

With the EDX-Mapping in Figure 5, the interface region of the coating was inspected where thermal-activated reactions occurred as interaction between the Cr and Ni of both the coating and substrate. An interdiffusion process, which depends on nucleation conditions, mobility of atoms, and build-up of driving forces, led to a growth of a Cr reaction layer in the coating next to the interface [30,31]. Furthermore, an Ni reaction layer formed in the coating above the Cr reaction layer. The present process is simplified to the assumption that interdiffusion between Cr und Ni took place in a binary system. Cr diffused from the coating towards the substrate forming separate Cr and Ni solid solution regions, the phenomena being displayed in Figure 6.

As shown in Figure 6 and indicated by the colored dashed lines at 900 °C, there is an equilibrium between a Cr-containing Ni-phase with ~38 wt-% Cr in Ni solid solution (cross point I) and an Ni-containing Cr-phase with ~5 wt-% Ni in Cr solid solution (cross point II). In between the cross points, a miscibility gap occurs due to the equilibrium state of the solid solutions showing that it is not possible to obtain a two-phase solid solution region by interdiffusion in a binary system [32]. This fact explains the well-defined border between the Cr- and Ni-rich phases from Figure 5.

While cooling down the system, a diffusion-controlled segregation process of elements can occur additionally to the single-layer growth process. However, this effect does not change the thickness of the present solid solution layers but rather its concentration profiles. The growth kinetics of the Ni reaction layer show the influence on the degradation of the substrate surface [33]. Therefore, slow kinetics are required to mitigate Ni degradation in the Ni-based (super-) alloys [33].

Although the coating is considered gas tight, selective oxidation occurs at the interface region. The presence of Al in the chemical composition of the investigated substrate favors the chemical processes to form locally isolated Al_2_O_3_ with oxygen (anions diffusion) from the former adjacent Cr oxides. Atmospheric molecular oxygen continuously supplied to the interface would lead to a selective oxidation of the elements Cr and Al under the formation of chromium oxide layers at the interface region [12].

Moreover, the formation of a stable intercrystalline TiN-phase can be also observed. The substrate contains 2–3 wt-% Ti that diffuses to the substrate surface during the heat treatment. There, it reacts with N to form TiN. In the present system, however, it is likely that the nitrogen has been soluted in the spraying powder or during the spraying process in the N_2_ gas stream, due to high-nitrogen affinity of Cr. This observation confirms the gas tightness of the coating, otherwise some Ti oxides or selective oxidation at the substrate surface might have occurred.

**Figure 7:**
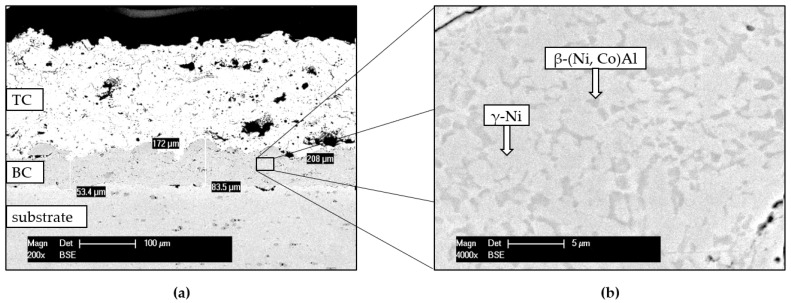
SEM/ cross-section micrographs of the as-sprayed TBC system (**a**) overview, (**b**) higher magnification of BC.

**Figure 8:**
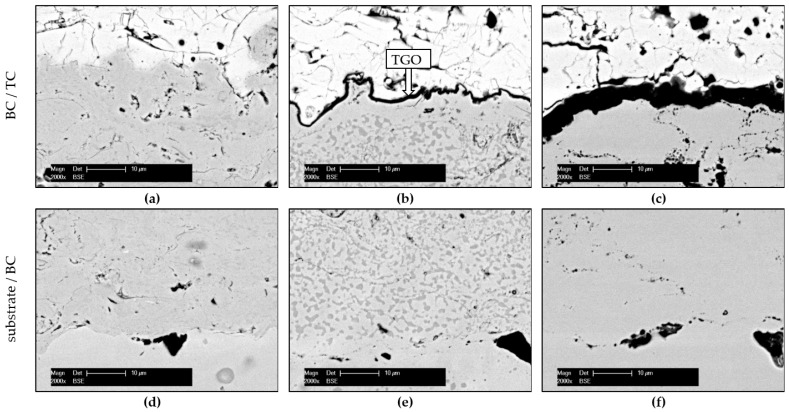
Cross-section SEM BSE micrographs of the TBC system (**a**,**d**) as-sprayed, (**b**,**e**) heat-treated (t_900°C_ = 10 h), and (**c**,**f**) isothermally oxidized (t_900°C_ = 1200 h).

Further experimental results concerning the characteristics of the as-sprayed and oxidized TBC system with the focus on the CoNiCrAlY BC are presented in Figure 7 and Figure 8.

The CoNiCrAlY BC deposition had an approximate final thickness of 70 µm, while the whole system (with YSZ-TC) reached a thickness of 250 µm, comparable to the thickness of the Cr-Cr_x_O_y_ coating. The primary role of the ceramic TC is to protect the base material from temperatures exceeding 1000 °C. Nevertheless, the interconnected pores and microcracks let the atmosphere penetrate the underlying metallic BC, causing oxidation of the coatings surface. The BC mainly consists of β-(Ni, Co)Al intermetallic phases and γ-Ni phases in solid solution state (Figure 7), but its microstructure might be even more complex [34]. The β phase contributes to the oxidation protection of the coating system, having a direct influence on the characteristics of the TGO formed along this interface (dark layer in Figure 8b,c).

Al was supplied towards the BC surface from the β phase and formed a stable and dense Al_2_O_3_ scale. Due to relatively slow oxidation kinetic, the formed TGO prevents further oxidation of other elements from the chemical composition of BC, thus protecting the substrate [35]. However, defect-like intersplat boundaries favors coatings inner oxidation, as already observed regarding the Cr-Cr_x_O_y_ coating.

Once the β-NiAl phase depletes with exposure time (comparing β phase distribution Figure 8e–f), a natural oxidation occurs with the other elements of the coating, which can cause the spallation of the YSZ top coat. However, the SEM micrographs presented in Figure 8f demonstrate that the investigated TBC system was able to protect the substrate against oxidation up to 1200 h. Neither spallation of the TBC, nor the Kirkendall effect in the BC occurred. This observation can be justified by the chosen oxidation parameters, under which the stress conditions might have been too moderate for the present TBC system.

### 3.2. Microhardness and Adhesion

**Figure 9:**
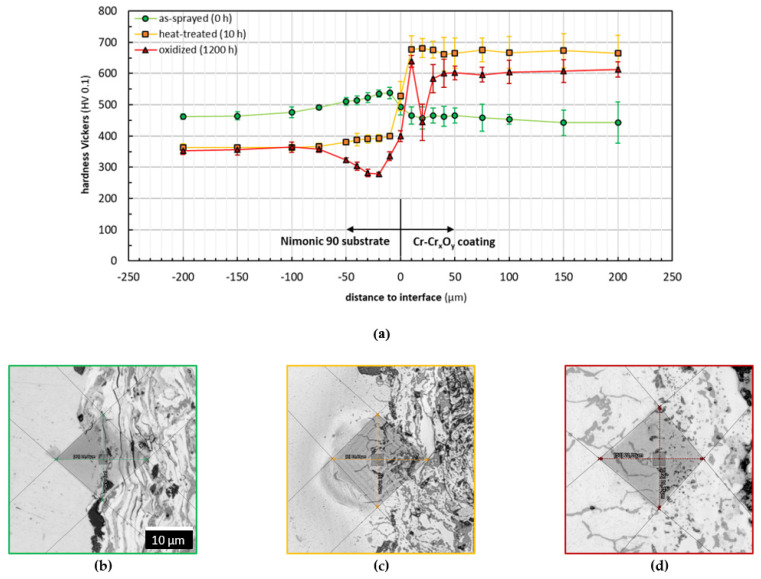
Evaluation of materials microhardness perpendicular to the interface substrate/Cr-Cr_x_O_y_ coatings: (**a**) Vickers microhardness profiles and corresponding cross-section CLSM micrographs of exemplary indents at the interface substrate/Cr-Cr_x_O_y_ coatings: (**b**) as-sprayed (green framed), (**c**) heat-treated (orange framed), and (**d**) isothermally oxidized (red framed).

**Figure 10:**
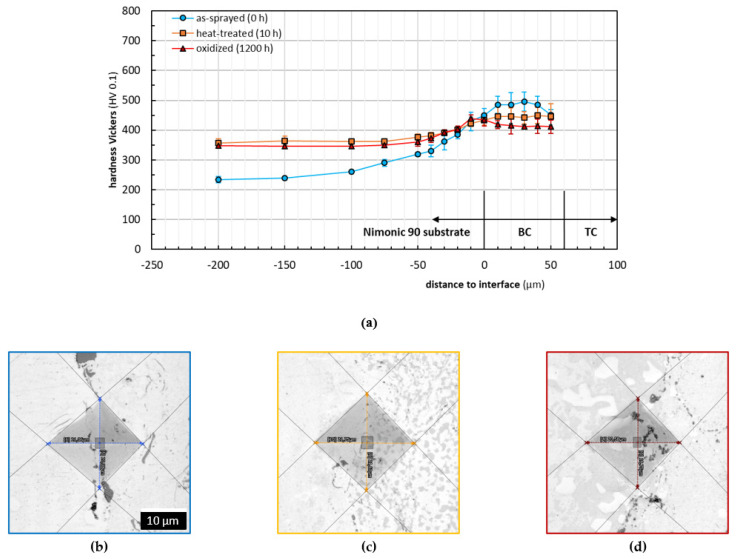
Evaluation of materials microhardness perpendicular to the interface substrate/BCs: (**a**) Vickers microhardness profiles and corresponding cross-section CLSM micrographs of exemplary indents at the interface substrate/BCs: (**b**) as-sprayed (blue framed), (**c**) heat-treated (orange framed), and (**d**) isothermally oxidized (red framed).

The microhardness distribution was determined by HV 0.1 Vickers indentations in the cross-section of the as-sprayed, heat-treated, and oxidized samples. To assure reproducibility, at least five indents were performed for each position in different distances to the substrate/coating interface. The generated indents were measured by means of CLSM with an accuracy of 0.1 µm and statistically evaluated. The obtained materials hardness profiles in dependence of the distance to the interface and corresponding cross-section CLSM micrographs of exemplary indents are presented in Figure 9 for the interface substrate/Cr-Cr_x_O_y_ coatings and in Figure 10 for the interface substrate/BCs in the TBC systems respectively.

As seen in Figure 9a, the microhardness of the substrate decreased during the heat treatment due to a softening of the former precipitation-hardened material. The hardness values remain comparable even over the prolonged dwell time at 900 °C. Due to compressive stresses caused by blasting hardening during the substrate preparation, the hardness increases towards the interface substrate/coating in the distance range of about −100 µm to −10 µm. The hardness increase is only present for the substrates in the as-sprayed and heat-treated states. Regarding the substrate in the oxidized state, the hardness decreases from −100 µm to −20 µm (beneath the interface) and increases towards the interface. This observation is mainly attributed to the described diffusion processes, which had been not analyzed in the study for the substrate in detail. However, the coatings alternation in hardness is influenced by many other factors. Regarding the as-sprayed coating interface in Figure 9b, the decrease in the hardness profile from the interface into the coating is strongly related to the coating microstructure and stress condition. It was found out that the microcracks of the as-sprayed coating were enlarged after indentation, proving the as-sprayed coating’s relatively poor adhesion to the substrate, as well as cohesion regarding its microstructure. For the as-sprayed coatings, the energy of the indentation loads mainly contributes to the crack propagation instead of plastically deforming the coatings, which leads to a lower microhardness of the as-sprayed coatings, contrary to the heat-treated samples.

This observation is not considered to be critical since the heat treatment leads to a better layer cohesion and a decrease in the crack susceptibility, which is mainly attributed to stress relief (promotion of compressive stresses), phase transformation, sintering, and elements diffusion [36,37,38].

Failures such as cracking or delamination are mostly prevented as a result of the strong metallurgical bonding formed at the interface region due to the thermal exposure associated with diffusion and interdiffusion processes.

Especially in the oxidized state at the interface region, the influence of diffusion processes and the reactive layer growth on the hardness are obvious (climax/low point in coatings profile). Decreases in the hardness measured for the oxidized coatings in comparison with those of the heat-treated ones is explained as a result of diffusion processes and a ceramic and metallic phase separation, combined with some changes concerning the degree of internal stress [36]. Nevertheless, the presence of the SiO_2_ could not be identified as a weakening factor in the coating.

The microhardness analysis for the TBC systems is displayed in Figure 10. It must be affirmed that the substrate used for the TBC systems was differently manufactured (softened) than the substrate of the Cr-Cr_x_O_y_ coatings. However, as shown in Figure 10a, once heat-treated, the substrate hardness adapts to the expected values shown in Figure 9a in the profile towards the interface. The hardening effect of the substrate preparation by alumina blasting can be observed, as well. Hardness values of the oxidized samples in the range of −100 µm to −10 µm did not change as significantly as those of the oxidized substrate in Figure 9a, which, in this case, is attributed to a smaller interdiffusion due to similar chemical compositions of the substrate and the BC. Regarding the coating hardness, the values decrease towards the ceramic TC in as-sprayed condition, contrary to the heat-treated and oxidized states, where they remain almost constant. An explanation might be the elastic/plastic mismatch stresses, which could occur near the BC/TC interface in the as-sprayed state. Due to the isothermal heat treatment, TCs were resintered. Furthermore, the formation of the TGO might lead to compressive stresses. Both microstructural changes have influence on the hardness towards the TC [39].

The BC chemical composition (mainly ductile materials), as well as optimized spraying parameters (dense microstructure), contributes to the reduction in the forming of microcracks when applying the same indentations loads, as shown in Figure 10b. Compared to the Cr-based system, smaller microcracks were visible at the interface substrate/BC. Due to similar chemical compositions of the substrate and BC, lower internal stresses occur when the coating cools down during the spraying process. Microcracks within the BC were only observed if the indentations were placed along intersplat boundaries. A lower crack tendency is due to a smaller quantity of intersplat boundaries, which mainly depends on the spraying powder/splat size, as well as the distribution of different adhering phases [40,41].

The hardness decrease in the heat-treated coatings mainly related to stress relief led to the conclusion that internal stresses were present in the as-sprayed state [42]. As in the Cr-based system, the heat treatment promotes a metallurgical bonding between substrate and coating and strengthens the coatings cohesion, since no microcracks were visible in the heat-treated and oxidized states. Sintering, as well as further oxidation at intersplat oxide stringers, might contribute to an improvement of coatings cohesion. After the samples were oxidized, the coatings’ hardness decreased slightly, which could be caused by a β depletion, as well as further stress relief. The latter one might have the main influence on the alteration of the BC microhardness.

## 4. Conclusions

Cr-Cr_x_O_y_ APS coatings and YSZ-CoNiCrAlY APS-HVOF thermal barrier coating systems were deposited on the NiCr20Co18Ti substrate. The thermal performance of the coatings was analyzed in the as-sprayed and heat-treated (for 10 h) and oxidized (for 1200 h) states at 900 °C. An analysis of the samples was performed with a focus on the alteration of microstructure, as well as of the microhardness and bond strength in the interface region.

The Cr-Cr_x_O_y_ coating consisted of Cr, metastable ferromagnetic CrO_2_, and stable Cr_2_O_3_ phases NiCr and SiO_2_. An isothermal heat treatment performed at 900 °C for 10 h showed that the present metastable CrO_2_ phase transformed into a Cr_2_O_3_ phase. A post treatment of the as-sprayed samples at the same temperature for either 10 h or 1200 h increased the oxide content from ≈27% up to ≈40%. The post treatment also initiated the formation of thermally grown Cr_2_O_3_ layers on the surfaces of the coatings. The prolonged oxidation of 1200 h led to a development of pores/voids under the layer due to diffusion of Cr ions outside of the coating. Moreover, as explained by the miscibility gap in the Cr-Ni binary diagram, well-defined Cr and Ni reaction layers were formed in the interface region during prolonged oxidation of the samples. The heat treatment was shown to increase the coatings’ microhardness. Additionally, the layers’ cohesions improved and therefore led to the decrease in the crack susceptibility due to stress relief, phase transformation, sintering, and element diffusion. The presence of the SiO_2_ carrier material was not seen as a weakening factor in the coating.

The YSZ-CoNiCrAlY system was analyzed with a focus on the bond coat since the top coat does not directly influence the oxidation behavior of the bond coat. The bond coat mainly consisted of a β-(Ni, Co)Al intermetallic phase embedded in a γ-Ni matrix. The interconnected pores and microcracks of YSZ top coating favored the penetration of normal atmosphere towards the bond coat, causing a surface oxidation of the bond-top-coating interface. The β-(Ni, Co)Al intermetallic phase promotes the growth of the thermally grown oxide layer, contributing to the protection of the coating system. Nevertheless, the β-NiAl depletes with exposure time. Regarding the microhardness of the coatings at different states, a smaller deviation could be noticed in comparison with that of the Cr-Cr_x_O_y_ coatings. The chemical composition and mechanical properties, which influence the adhesion and cohesion, are discussed as possible reasons for the achieved coatings properties.

The results show a significant influence of the sample’s dwell time under a defined isothermal load on the coating’s thermal behavior (microstructure, mechanical properties) and thus on its protection function. As demonstrated, although the individual coating’s thermal behavior is different, in both types of coatings, the substrate’s oxidation protection could be observed. This finding supports the possible usage of Cr-Cr_x_O_y_ coatings under the mentioned conditions and loads as a promising alternative to the state of the art systems for oxidation protection. Further work is planned to analyze the wear and erosion behavior of the coatings in different environments.

## Data Availability

The data reported in this study are available from the authors upon request.
